# Supplementary material for: Assessing the importance of demographic risk factors across two waves of SARS-CoV-2 using fine-scale case data
Source: PLoS Comput Biol. 2023 Nov 27;19(11):e1011611. doi: 10.1371/journal.pcbi.1011611 (PMC10703279; doi:10.1371/journal.pcbi.1011611)
Supplement: S1 Text — A. Supplementary plots for the time evolution of cases across the Delta and Omicron waves. B. Additional methodology details; hyperparameter selection, detailed description of all explanatory variables. C. Map view of population distribution of Scotland, and model residuals for Omicron model. D. Plots for explanatory variable Importance; node purity, accuracy loss on variable permutation. E. Additional details on lateral flow testing frequency, broken down by sex and deprivation quintile. (PDF) [file pcbi.1011611.s001.pdf]

# S1 Text

*Supporting information: Assessing the importance of demographic risk factors across two waves of SARS-CoV-2 using fine-scale case data*

Anthony J. Wood, Aeron R. Sanchez, Paul R. Bessell, Rebecca Wightman, Rowland R. Kao

# A Supplementary plots for time evolution of cases

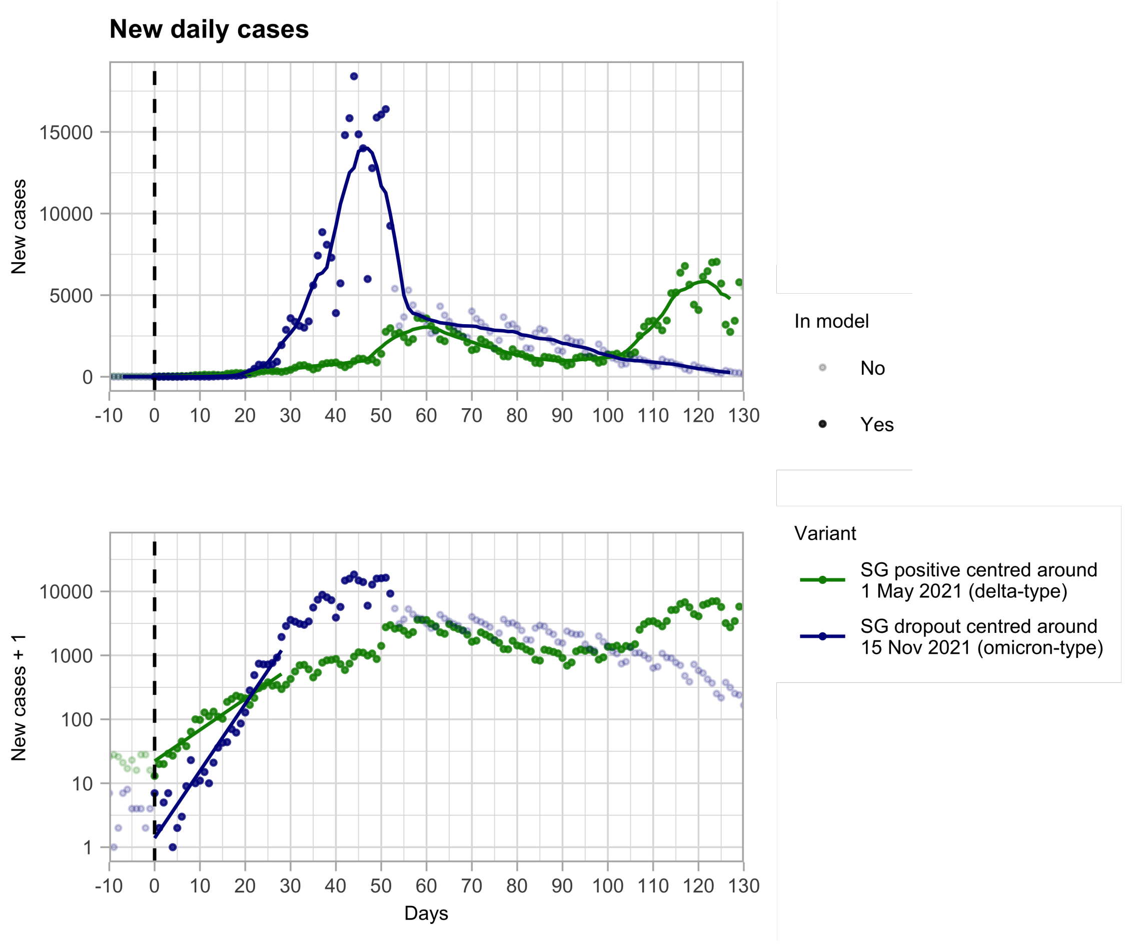


Figure A: Timeseries of the initial outbreaks of the Delta and Omicron variants in terms of newly reported cases. The gradient of the linear regression (straight line) of the early trajectory of log(new cases + 1) is inversely proportional to the case doubling time.

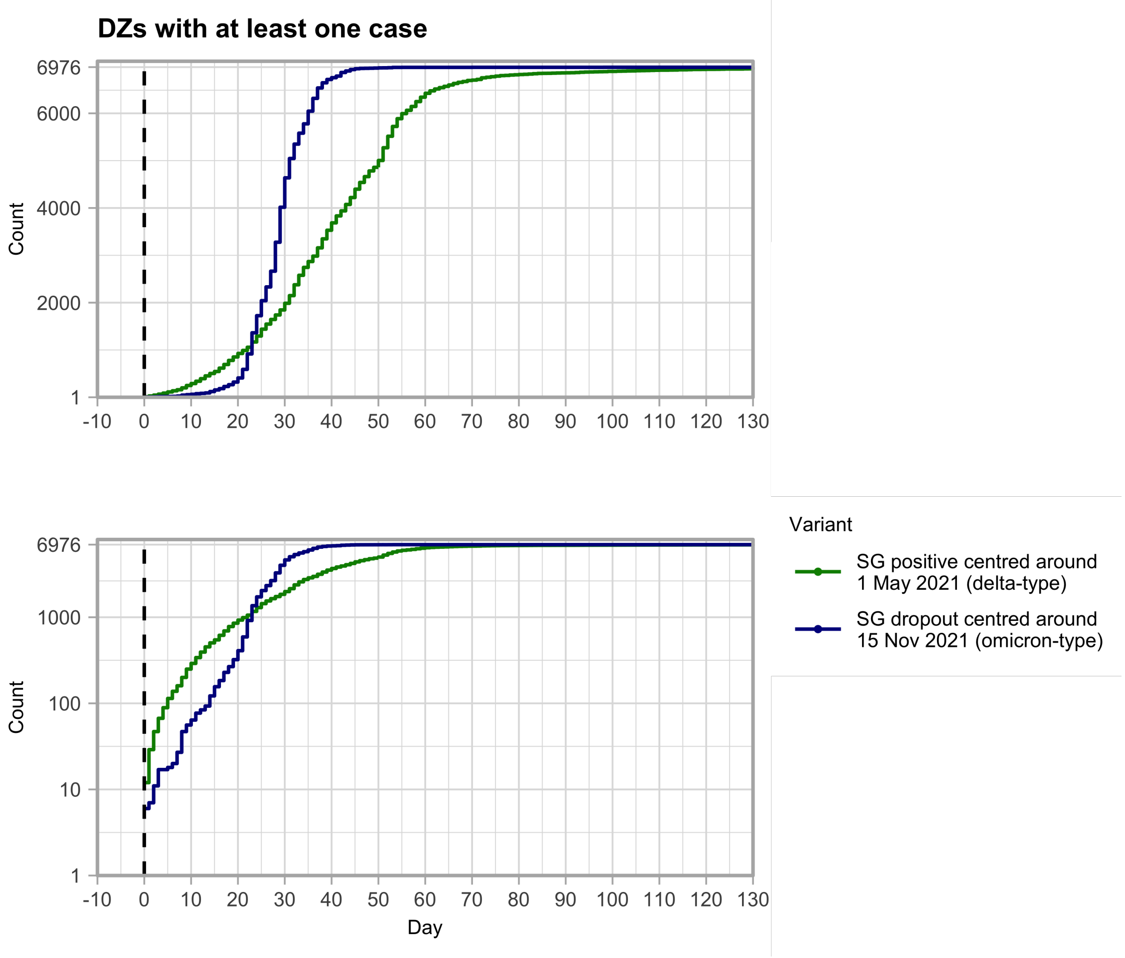


Figure B: Timeseries of the initial outbreaks of the Delta and Omicron variants in terms of the cumulative number of DZs to have reported at least one case associated with the variant of interest.

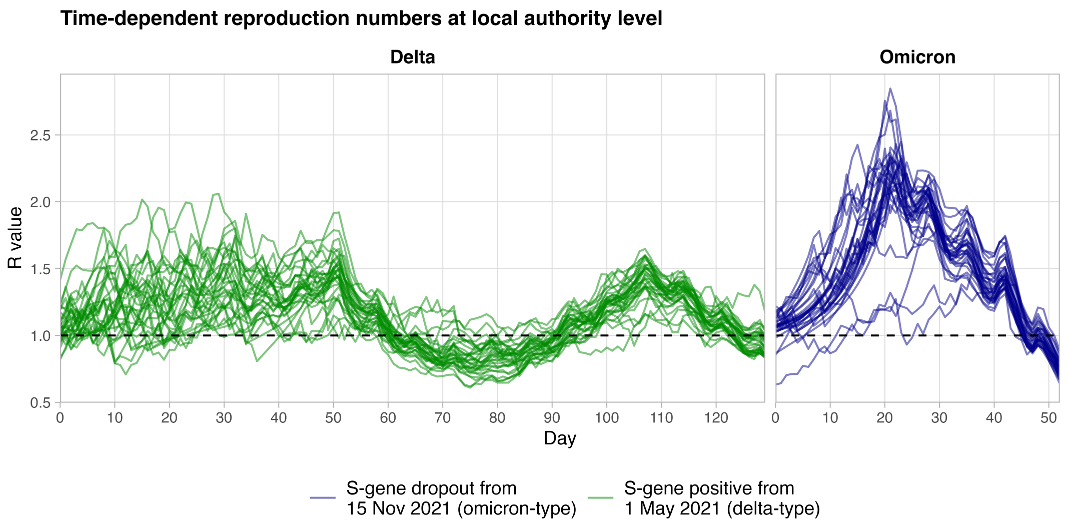


Figure C: Time-dependent reproduction numbers for the Delta (left) and Omicron waves (right), over each of the 32 individual local authorities.

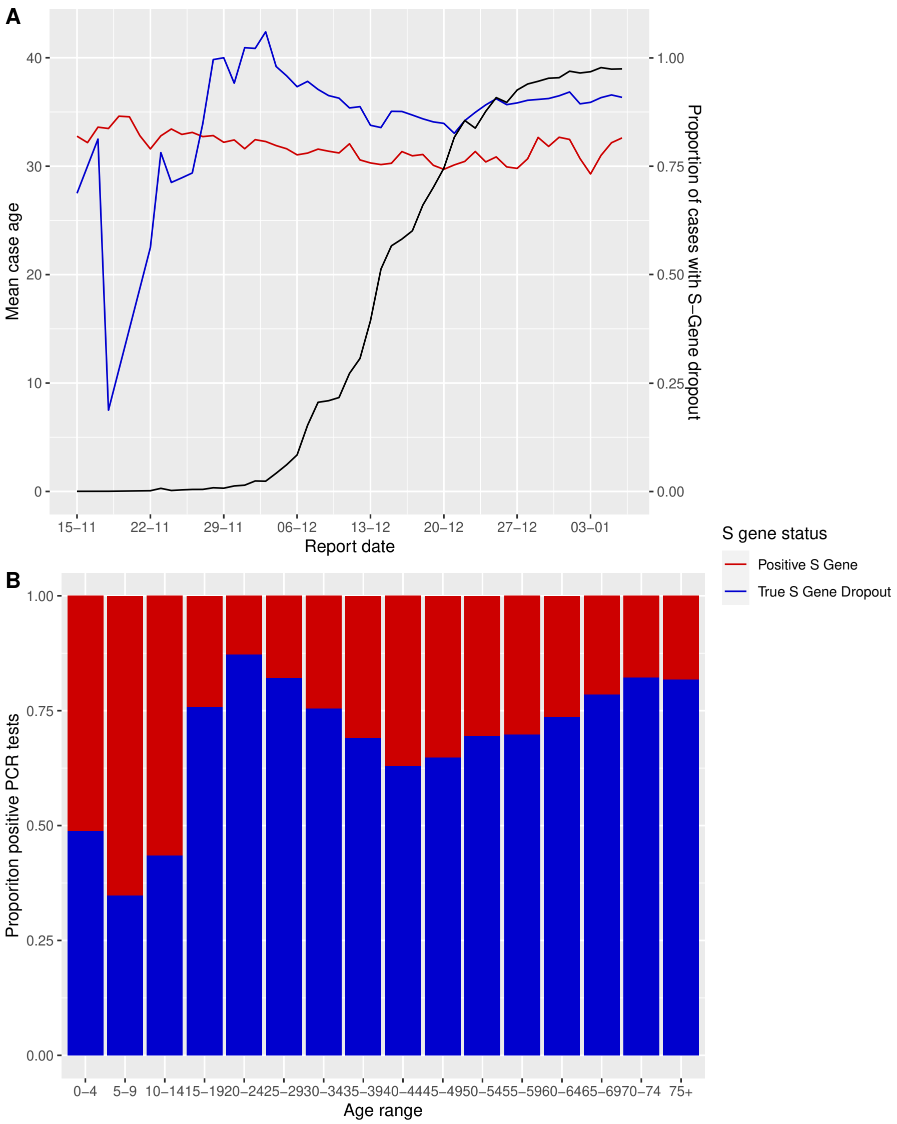


Figure D: PCR positive cases over the period 15$^{th}$ November 2021 to 6$^{th}$ January 2022 that were S-gene dropout or true S-gene positive. (A) Daily mean case age for the two definite PCR S-gene outcomes (blue and red lines) against the proportion of the daily cases that were true S-gene dropout (presumed Omicron-type). (B) the proportion of the cases over the period by 5-year age bracket.

# B Additional methodology details

## B.1 Model hyperparameters

The random forest regression model is fit in *R* version 4.1.0 [1], using the *randomForest* package [2] (version 4.6-14), and ALEs analysed using the *ALEPlot* package [3] (version 1.1).

From 6,976 DZs, 2 sexes, 16 age ranges, and 3 prior case states, there were a total of 669,696 cohorts (of which a fraction will have population zero and are excluded). Cohorts from 90% of DZs were used for the fit, with 10% reserved to test model performance against data it explicitly did not fit. The fit was made to $\sqrt{\text{cases} + 1}$. The RF comprised 500 trees, with cohorts sampled for building each tree weighted by population. 5 variables were tested at each split, and each tree had a maximum of 30,000 terminal nodes, with a minimum node size of 300.

## B.2 Explanatory variables used in random forest regression model

The models described in Section 4.3 are informed with the following data, first at cohort resolution:

- *Age range* (five-year windows: $[0-4]$, $[5-9]$, ..., $[70-74]$, [75+]), using the numeric intermediate values $2, 7, \ldots 72$, and 75 for the 75+ category;
- *Sex*;
- *Prior case status*: the time of the last reported case, broken into three categories: never tested positive before, last tested positive in the 6 months prior to the first day of the outbreak, last tested positive over 6 months prior;
- *Cohort population* (derived using historical testing data for those testing positive before, and estimated populations as of mid-2020 collated by the National Records of Scotland [4], for the remainder that had not tested positive before).

At age/sex/DZ resolution, we then include:

- COVID-19 *vaccination uptake* (eDRIS) (see also S1 Section B.3);
- *Ethnicity* (% population belonging to a minority ethnicity), as per the most recent Scottish census data (2011);
- The per-population, time-aggregated number of *negative LFD tests* reported in that period.

Finally included are the following at DZ resolution or broader:

- Measures of DZ-level deprivation (obtained from Scottish census data, and the 2020 *Scottish Index of Multiple Deprivation* [5]);
- *Local outbreak duration*: the difference between the final date of the period studied, and the date the variant was first detected in that cohort's corresponding *intermediate zone* (*IZ*). An IZ typically contains 4–6 DZs, and 3,000–5,000 individuals, with this granularity chosen to give a reasonable proxy for when the variant was seeded locally;
- *Student population* (% population being a full-time student aged 18 or over), also per 2011 census data;
- *Population density*, at IZ-level;
- *S-gene coverage* (the proportion of cases with an accompanying S-gene result, required to associate a likely variant) at IZ level. S-gene coverage was 90% overall across mainland Scotland (per eDRIS data), but significantly lower in the LAs of Orkney Islands, Shetland Islands and Na h-Eileanan Siar (74%, 20% and 23% respectively).

The measures of DZ-level deprivation included are [6]:

- *Drive time from GP*: Average drive time to a GP surgery in minutes;
- *Public transport time to GP*: Public transport travel time to a GP surgery in minutes;
- *% Income deprived*: Proportion of individuals in receipt of income support payments, such as Job Seekers Allowance;
- *% Employment deprived*: Proportion of working age population claiming employment-related payments, such as Incapacity Benefit;
- *Standardised mortality ratio*: Age/sex-standardised mortality rate as compared to the overall population;
- *Comparative illness factor*: Proportion of individuals claiming from a variety of illness and disability-related payments as compared to the overall population;
- *Drug-related hospitalisation ratio*: Rate of hospitalisations relating to drug use, as compared to the overall population;
- *Alcohol-related hospitalisation ratio*: Rate of hospitalisations relating to alcohol use, as compared to the overall population;
- *Crime rate*: Rate of recorded crimes per population;
- *Attendance*: Percentage of pupils with school attendance of over 90%;
- *Attainment*: Measure for average attainment of school leavers from 2015–2018;
- *Ratio working age with no qualifications*: Proportion of working age people with no qualifications, as compared to the overall population.

We do not use data on *PCR* negative tests. In the Omicron wave PCR positivity peaked at 30% (per eDRIS data), with testing capacity being reached (resulting in a policy change on 5$^{\text{th}}$ January 2022 removing the need for a confirmatory PCR after an LFD positive [7]). Thus with this "ceiling" capacity being reached, we exclude negative PCR tests as a poorer proxy for propensity to test as compared to LFD negatives, and being too closely related to overall cases (requiring an S-gene sequenced positive PCR test).

## B.3 Vaccination uptake as an explanatory variable

Scotland's COVID-19 vaccination programme began on December 8$^{\text{th}}$ 2020, with initial priority given to healthcare workers, the elderly and those otherwise especially vulnerable to COVID-19, then generally by decreasing age [8]. All first doses had been offered and administered to willing adults by 18$^{\text{th}}$ July 2021 [9], with rates of first dose administration declining thereafter. By 15$^{\text{th}}$ November 2021, then, the first dose date may have differed between two individuals by up to 11 months. This likely led to substantial variation in protection offered by the first dose at the time of the Omicron wave, given both evidence of efficacy waning over timescales of six months, and high rates of breakthrough for Omicron against vaccines originally designed against earlier "wild-type" SARS-CoV-2 lineages, particularly for non-mRNA vaccines [10, 11, 12]. This, combined with high uncertainty in the cohort-level population denominator used to determine uptake, leads us to exclude first and second dose uptake (being highly correlated with first dose uptake) as an explanatory variable for Omicron cases. We do, however, include third/booster dose uptake, as the proportion receiving a first dose to have *returned* for a third/booster dose by 15$^{\text{th}}$ November 2021 (and zero if nobody in the cohort had yet received a first dose). This definition eliminates uncertainty in the underlying population. Prior to the detection of Omicron, those aged 50+ or otherwise vulnerable to COVID-19 were due to be offered a third or booster dose, twelve weeks after their second [13]. The booster programme began on September 20$^{\text{th}}$ 2021, and a snapshot on 15$^{\text{th}}$ November 2021 shows substantial variation between different cohorts, particularly by age.

With these doses being delivered more recently, as well as evidence of this dose proving more protective against Omicron [10, 14], we include this definition of third/booster dose uptake as a reasonable proxy for vaccine-induced protection against Omicron at the time.

The initial Delta wave occurred while the bulk of first and second doses were still being administered, thus we include second dose uptake on 1st May 2021 as an explanatory variable, as the proportion of individuals that had returned for a second dose, having received a first (and zero, if nobody in the cohort had yet received their first dose).

# C Map views of population distribution, model residuals

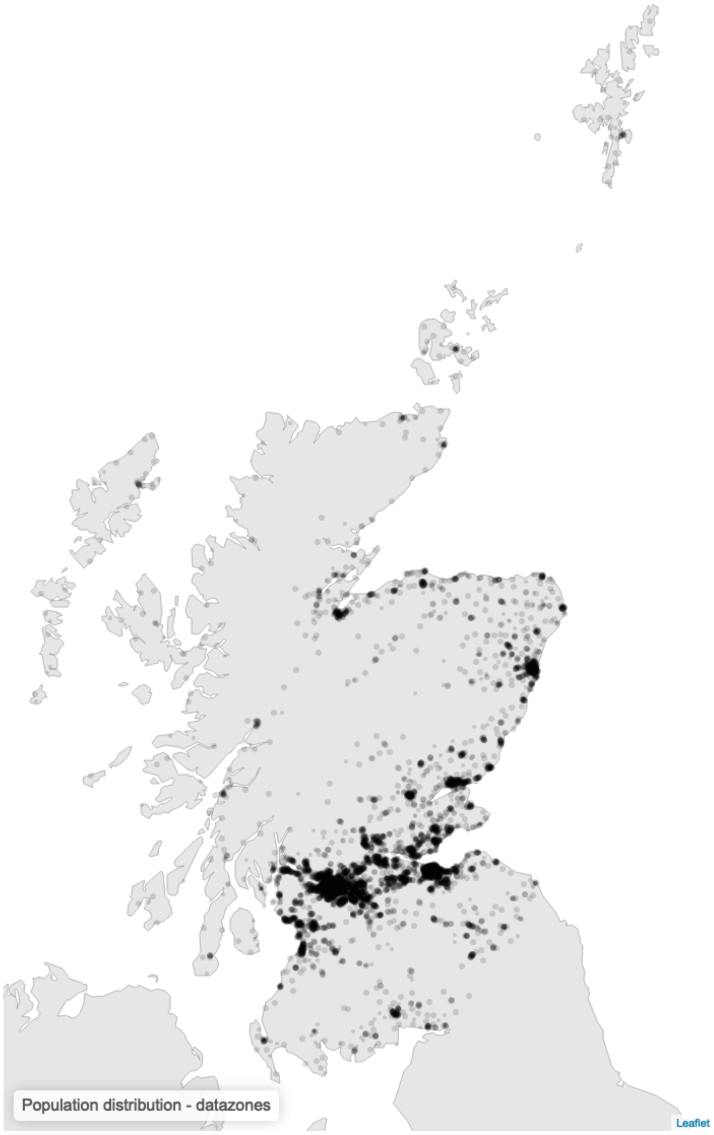


Figure E: Distribution of population in Scotland. Each point indicates the population-weighted centroid of a datazone (DZ) [15] of which there are 6,976 in total, with each representing a population of approximately 500-1,000 individuals. Base maps obtained from Natural Earth [16].

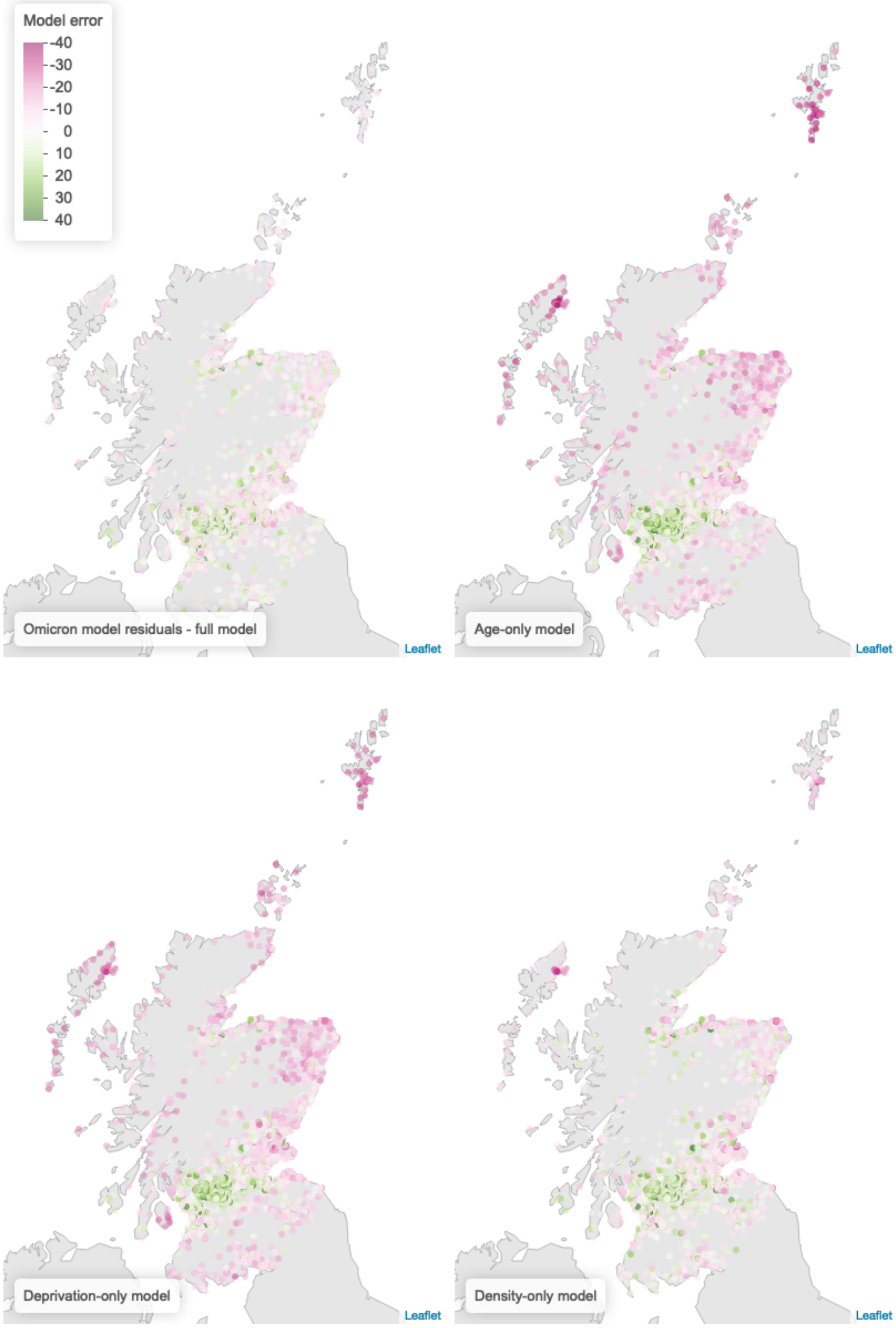


Figure F: Comparing residuals for the distribution of Omicron cases, between the full model (top left), and the reduced models informed by population and one of age, deprivation and population density respectively. The colour scale indicates the DZ-level model error (model estimate - data), where purple points indicate DZs where the model overestimated the number of cases, and green points indicate where the model underestimated cases. Base maps obtained from Natural Earth [16].

# D Random forest variable importance

Fig G shows variable importance measures extracted from the *RandomForest* function. Age, population and prior case status have much higher node purity (Fig G, top) than the other variables, indicating that splits in individual trees using values of these variables in particular are characteristically more "effective" at separating cohorts with differing numbers of cases. Fig G, bottom, then shows random permutation of each of the variables results in appreciable increase in fit error, confirming that this larger collection of variables are important to explain finer patterns in the data.

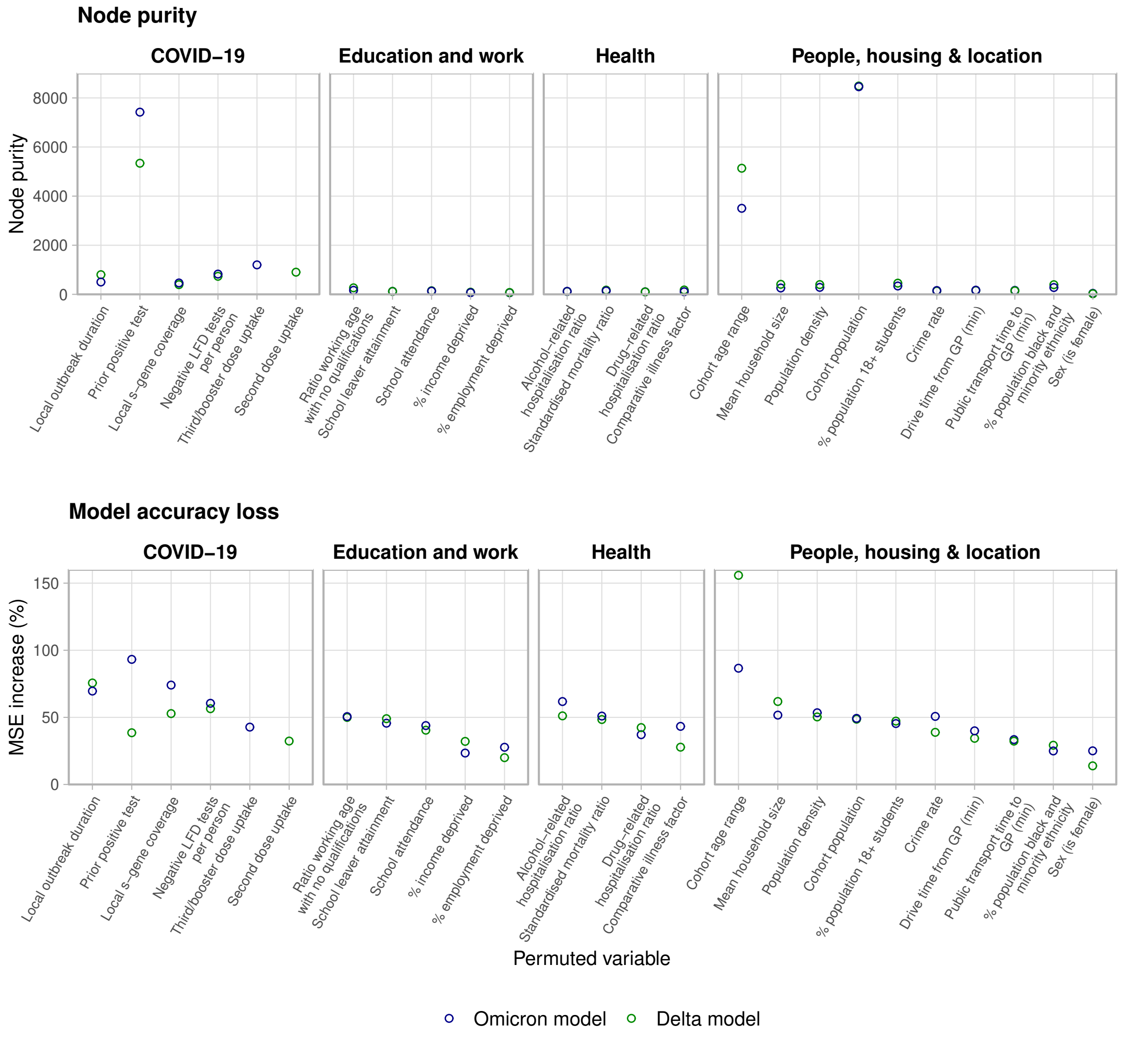


Figure G: Feature importance outputs form the random forest regression models. Top: Node purity. Bottom: Explanatory variable mean squared error (MSE) increase on random permutation: for variable $i$, the increase in MSE on data not trained in each tree, if the entries of $i$ were instead randomly permuted.

# E Frequency of lateral flow testing by sex, deprivation quintile

| | | Population | LFD tests | Tests per person | Positive LFD tests | Positivity |
|---|---|---|---|---|---|---|
| Total | | 5,466,000 | 29,508,794 | 5.40 | 1,123,210 | 3.81% |
| Sex | F | 2,800,788 | 19,639,047 | 7.01 | 647,529 | 3.30% |
| | M | 2,665,212 | 9,869,747 | 3.70 | 475,681 | 4.82% |
| Deprivation quintile (1: most deprived) | 1 | 1,057,767 | 3,827,970 | 3.62 | 176,600 | 4.61% |
| | 2 | 1,057,929 | 4,992,257 | 4.72 | 201,148 | 4.03% |
| | 3 | 1,077,589 | 6,023,840 | 5.59 | 220,258 | 3.66% |
| | 4 | 1,140,448 | 7,134,794 | 6.26 | 256,101 | 3.59% |
| | 5 | 1,132,267 | 7,529,933 | 6.65 | 269,103 | 3.57% |

Table A: Summary statistics of lateral flow device (LFD) tests reported in Scotland from July 2020 to February 2023, broken down by sex, and deprivation quintile of the residing datazone of individuals as ranked by the 2020 Scottish Index of Multiple Deprivation, where the most deprived datazones are in quintile 1. The test positivity is the proportion of all tests of any result that were reported as positive.

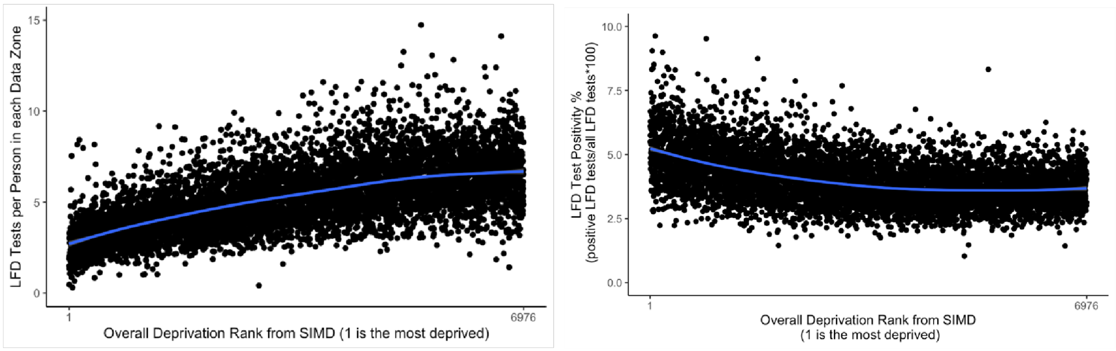


Figure H: Lateral flow testing from July 2020 to February 2023 by datazone, ranked by deprivation per the 2020 Scottish Index of Multiple Deprivation, where the rank 1 is the datazone ranked as most deprived. Left: the number of LFD tests reported per person in each datazone. Right: the LFD test positivity, defined as the proportion of all reported LFD tests to have been positive.

# References


[1] R Core Team. R: A Language and Environment for Statistical Computing. Vienna, Austria; 2022. Available from: https://www.R-project.org/.

[2] Liaw A, Wiener M, et al. Classification and regression by randomForest. R news. 2002;2(3):18–22.

[3] Apley D, Apley MD. Package 'ALEPlot'. 2018;.

[4] National Records of Scotland. Mid-2020 Small Area Population Estimates for 2011 Data Zones;. Available from https://www.nrscotland.gov.uk/statistics-and-data/statistics/statistics-by-theme/population/population-estimates/small-area-population-estimates-2011-data-zone-based/mid-2020/ (last accessed 15/08/2023).

[5] The Scottish Government. Scottish Index of Multiple Deprivation 2020;. Available from https://www.gov.scot/publications/scottish-index-of-multiple-deprivation-2020v2-indicator-data/ (last accessed 15/08/2023).

[6] The Scottish Government. SIMD 2020 Technical Notes;. Available from https://www.gov.scot/publications/simd-2020-technical-notes/ (last accessed 15/08/2023).

[7] The Scottish Government. Self-Isolation and testing changes;. Available from https://www.gov.scot/news/self-isolation-and-testing-changes/ (last accessed 15/08/2023).

[8] The Scottish Government. Coronavirus (COVID-19): vaccine deployment plan 2021;. Available from https://www.gov.scot/publications/coronavirus-covid-19-vaccine-deployment-plan-2021/ (last accessed 15/08/2023).

[9] The Scottish Government. Major milestone in vaccination programme;. Available from https://www.gov.scot/news/major-milestone-in-vaccination-programme/ (last accessed 15/08/2023).

[10] Andrews N, Stowe J, Kirsebom F, Toffa S, Rickeard T, Gallagher E, et al. Covid-19 vaccine effectiveness against the omicron (B. 1.1. 529) variant. New England Journal of Medicine. 2022;.

[11] Cele S, Jackson L, Khoury DS, Khan K, Moyo-Gwete T, Tegally H, et al. Omicron extensively but incompletely escapes Pfizer BNT162b2 neutralization. Nature. 2022;602(7898):654–656.

[12] Vasileiou E, Simpson CR, Shi T, Kerr S, Agrawal U, Akbari A, et al. Interim findings from first-dose mass COVID-19 vaccination roll-out and COVID-19 hospital admissions in Scotland: a national prospective cohort study. The Lancet. 2021;397(10285):1646–1657.

[13] The Cabinet Secretary for Health, Care S. Scotland's autumn/winter vaccination strategy 2021;. Available from https://www.gov.scot/publications/scotlands-autumn-winter-vaccination-strategy-2021/ (last accessed 15/08/2023).

[14] Sheikh A, Kerr S, Woolhouse M, McMenamin J, Robertson C. Severity of Omicron variant of concern and vaccine effectiveness against symptomatic disease: national cohort with nested test negative design study in Scotland. 2021;.

[15] The Scottish Government. Data Zone Centroids 2011;. Available from https://spatialdata.gov.scot/geonetwork/srv/api/records/8f370479-5e3d-450b-9064-4a33274f1a52 (last accessed 11/09/2023).

[16] Natural Earth. Terms of Use;. Available from https://www.naturalearthdata.com/about/terms-of-use/ (last accessed 19/09/2023).
