# Supplementary material for: Assessing the importance of demographic risk factors across two waves of SARS-CoV-2 using fine-scale case data
Source: PLoS Comput Biol. 2023 Nov 27;19(11):e1011611. doi: 10.1371/journal.pcbi.1011611 (PMC10703279; doi:10.1371/journal.pcbi.1011611)
Supplement: Attachment — Submitted filename: Response to Reviewers.pdf [file pcbi.1011611.s002.pdf]

Feedback from referees and responses

September 19 2023

Initial review letter from editor

Dear Professor Kao,

Thank you very much for submitting your manuscript "Assessing the importance of demographic risk factors across two waves of SARS-CoV-2 using fine-scale case data" for consideration at PLOS Computational Biology.

As with all papers reviewed by the journal, your manuscript was reviewed by members of the editorial board and by several independent reviewers. In light of the reviews (below this email), we would like to invite the resubmission of a significantly-revised version that takes into account the reviewers' comments.

We cannot make any decision about publication until we have seen the revised manuscript and your response to the reviewers' comments. Your revised manuscript is also likely to be sent to reviewers for further evaluation.

When you are ready to resubmit, please upload the following:

1. A letter containing a detailed list of your responses to the review comments and a description of the changes you have made in the manuscript. Please note while forming your response, if your article is accepted, you may have the opportunity to make the peer review history publicly available. The record will include editor decision letters (with reviews) and your responses to reviewer comments. If eligible, we will contact you to opt in or out.

2. Two versions of the revised manuscript: one with either highlights or tracked changes denoting where the text has been changed; the other a clean version (uploaded as the manuscript file).

Important additional instructions are given below your reviewer comments.

- Figure Files: While revising your submission, please upload your figure files to the Preflight Analysis and Conversion Engine (PACE) digital diagnostic tool, https://pacev2.apexcovantage.com. PACE helps ensure that figures meet PLOS requirements. To use PACE, you must first register as a user. Then, login and navigate to the UPLOAD tab, where you will find detailed instructions on how to use the tool. If you encounter any issues or have any questions when using PACE, please email us at figures@plos.org.

- Data Requirements: Please note that, as a condition of publication, PLOS' data policy requires that you make available all data used to draw the conclusions outlined in your manuscript. Data must be deposited in an appropriate repository, included within the body of the manuscript, or uploaded as supporting information. This includes all numerical values that were used to generate graphs, histograms etc.. For an example in PLOS Biology see here: `http://www.plosbiology.org/article/info\%3Adoi\%2F10.1371\%2Fjournal.pbio.1001908#s5`.

- Reproducibility: To enhance the reproducibility of your results, we recommend that you deposit your laboratory protocols in protocols.io, where a protocol can be assigned its own identifier (DOI) such that it can be cited independently in the future. Additionally, PLOS ONE offers an option to publish peer-reviewed clinical study protocols. Read more information on sharing protocols at `https://plos.org/protocols?utm_medium=editorial-email&utm_source=authorletters&utm_campaign=protocols`.

Please prepare and submit your revised manuscript within 60 days. If you anticipate any delay, please let us know the expected resubmission date by replying to this email. Please note that revised

manuscripts received after the 60-day due date may require evaluation and peer review similar to newly submitted manuscripts.

Thank you again for your submission. We hope that our editorial process has been constructive so far, and we welcome your feedback at any time. Please don't hesitate to contact us if you have any questions or comments.

Responses to reviewers

We thank the reviewers for their helpful feedback and suggestions. In response to their comments we have we enclose a significantly revised version of the manuscript, as well as a document highlighting changes against the original manuscript, and the original manuscript.

As the changes from the original manuscript have been substantial and structural, the enclosed "changes" document may be difficult to follow. Below is a summary of major changes to the manuscript:

- Significant revision to the *Introduction* section to present more clearly the context of COVID-19 in Scotland in the period our work focuses on, our aims with the work and how it builds on the existing literature.
- Addition of several more references to the literature on risk factors for COVID-19 cases and severe outcomes.
- Significant revision to the *Results* section to remove text more suitable for the *Discussion* or *Materials and methods* sections.
- Removal of data relating to hospitalisation. In hindsight it was decided that this was confusing the main narrative of the manuscript (understanding the distribution of cases) without substantially adding to the analysis or the existing literature.
- Significant revision to the *Discussion* section to clarify the main findings of the paper, particularly in the context of accumulated local effects from the model, as well as model weaknesses.
- Additional work with respect to lateral flow testing frequency across demographics, and implications on the overall distribution of cases.
- Significant expansion and restructuring of the *Materials and Methods* section to describe how the data were prepared, and methods used in different sections of the paper. New section describing the Moran's I statistic.
- Revision of data availability statement to detail our access to the data, and how other researchers may gain access to it.
- Map figures revised with open-source basemaps (Natural Earth).

Finally we note the addition of Rebecca Wightman as a co-author on the paper, who performed the additional analysis on lateral flow testing frequency.

Please find below specific item-by-item responses to the reviewers' feedback, with corresponding changes in the manuscript.

Sincerely,

Anthony Wood (on behalf of all the authors)

Reviewer 1

This manuscript describes an analysis of COVID-19 testing data aggregated across local geographical units in Scotland, between two time periods with different dominant variants. Overall I feel that the manuscript lacks focus - it is not clear what the aim of the work is and how this fits into the existing literature. The methods section does not give sufficient information to understand what analyses were conducted, how they were conducted, and to address what questions. Some conclusions are drawn which are not supported by what is presented in the paper, or by other cited sources. The conclusions which are related to these specific results don't appear to add to what is already known on the subject.

Introduction

- Little mention of related literature

  Authors: We have added several more references to related studies, and clarify in the *Introduction* section how our work sits relative to the exisitng literature.

- The objective is not clear. Background paragraphs are broad and non-specific to this analysis. Is the interest in identifying risk factors for transmission/infection/mortality?

  Authors: We revised the *Introduction* section to make our aim more clear to the reader from the outset. Our primary aims are to investigate a) what the risk factors for COVID-19 were in Scotland, and b) how or whether they changed over time, to serve as an indicator for the longer-term patterns of infection.

- Mentions heterogeneity and "finely-grained data" and I assume the authors mean with respect to space, but this is not explicit.

  Authors: This is with respect to space (using the datazone identifier to pinpoint location, and then associate additional data associated with that location. We clarify this in the *Introduction* and later in *Materials and Methods*..

- The authors should better describe the existing literature that has explored these kind of data/patterns, and highlight how what you're doing is different/complementary.

  Authors: We now extend and clarify this in the main text. For the benefit of the reviewers we further detail this here:

  Our work builds upon the existing literature in three broad ways: 1) Our analysis is at a very fine datazone level which has not been covered in the literature before at this comprehensive national level. Studies in the existing literature are typically at much coarser scale, or over a more limited population, e.g., a district or town. Scotland has a very diverse geography (with a mix of dense urban and very rural communities and a large range of deprivation), and presenting a single model to describe the full distribution of cases is an important challenge; 2) The period we study is one where NPIs were mostly lifted (especially so for Omicron) but regular testing still in place, thus the trends here provide an important contrast as compared with cases in the first wave or prior to vaccination; 3) We include testing *frequency* as an explanatory variable, and discuss willingness to test in the context of how it may "skew" the distribution of cases to demographics that simply tested more.

  We have now added a paragraph to the *Introduction* section that places our work in comparison to existing literature, addressing the three above points.

- Could give more context about the pandemic in Scotland specifically.

  Authors: We have added a paragraph to the *Introduction* section describing the progression of the epidemic in Scotland.

- The introduction mostly describes the analysis approach, rather than providing relevant context and justifying the question to be addressed/the approach taken to address it.

Authors: We have made a substantial revision to the *Introduction* approach which clarifies our motivation, with methods/technical details reserved for the *Data and methods* section. On this point from the reviewer specifically, we have removed the paragraph discussing the analysis approach (beginning *"To compare the two waves, we fit a machine learning model..."*, and provided further motivation and context in earlier paragraphs.

Data and Methods

- Unclear why the data were split into "cohorts".

  Authors: A "cohort" (i.e. a group of individuals with the same sex, age range and residing datazone) is effecively the highest resolution we can distinguish using the cases data. Thus we design the model to fit cases at this level to maximise the granularity of the data being used. We have defined this more clearly in the final paragraph of section *Preparation of case data*, with an example of a cohort.

- What is the time scale for last testing positive? By month, week?

  Authors: We group individuals by a) never tested positive before, b) tested positive in the prior 6 months and c) tested positive over 6 months prior. This is specified in section *Preparation of case data*, and we point the reader to this in the *Model* section.

- If observations are already split into cohorts based on age/sex, is it appropriate to then use age/sex again as explanatory variables? Why necessary to do both?

  Authors: To give a brief example, if one took two cohorts from the same datazone but different age and sex (e.g. [50-55, M, DZ0012345], and [20-24, F, DZ0012345]), then all variables associated with geography/deprivation would be the same. However, the data suggest that case rates between these cohorts are likely to be quite different. Thus with the DZs equal, our analysis indicates that cohort age is an important variable in explaining this variation (as well as differences in vaccine uptake/testing rates, as we consider), though we do not claim this to be by any means causative.

- There should be a clear model specification included here, explicitly defining the outcome(s) and predictors.

  Authors: We have compartmentalised *Data and methods* to add a *Model* section, describing the model specification and variables used. Further parameter details and hyperparameters are given in the Supplementary Information, section *"Additional methodology details"*.

- The analysis approach should be described fully, referring to all variations of the model that were fit (results mentions a "full" model as well as several "univariate" but these are not defined).

  Authors: Referring to the above point, we have added to the *Data and methods* section to make the approach clearer. We now describe the "univariate" (now "reduced") model specifications in the *Random forest model* section as well.

- What was different between the "case distribution" and "spatial" analysis?

  Authors: We consider the "case distribution" to be how cases vary across a broad spectrum of variables (with age, deprivation, urban/rural classification etc.) and not explicitly space. A "spatial" analysis more explicitly considers case variation/residuals with respect to the physical locations of DZs (e.g. in the context of the Moran's I analysis). For clarity we have now replaced "spatial" with "geographical" in parts of the main text where we feel it is more appropriate.

- Descriptive analyses should also be explained here (the results section talks about "regression on log10(cases)", doubling times, Rt, a hypothesis test of age between variants).

  Authors: We have written new sections in *Data and methods* that describe how we calculate the doubling time and R_t (moving this from the Supplementary Information).

- Reading this section it isn't apparent what question is being addressed. Is the aim to compare between periods or variants? Or to compare explanatory power of different variables?

  Authors: The restructuring of the *Model* section and rewriting of the *Introduction* now makes our aims clearer to the reader.

  Both of these are of interest (what the dominant risk factors are, how they differed between waves), as we now specify in the opening of the *Model* section. It is more difficult to disentangle *why* those risk factors changed, between changes in the virus strain and imposed control measures. We have now stated in the *Discussion*.

  For context it is worth noting that this analysis was initially formed as the Omicron outbreak was taking place, where the main concern was in finding risk factors rather than the underlying causes.

- Why the need to have similar number of cases in each time period?

  Authors: There was no strict requirement to have a similar number of cases for each wave. Our interest is not necessarily in the volume of cases reported for each variant/wave, rather how those cases were distributed amongst different demographics. Analysing a similar volume of cases in each wave makes it easier to illustrate and discuss differences between the two variants (e.g., Fig. 1 is represented as cases / 1,000).

- Are you excluding cases caused by other variants in the same periods?

  Authors: We are; this is now stated in the *Preparation of case data* section.

Results

- Meaning of S-gene dropout/positive and a timeline of the different variants should be briefly explained.

  Authors: We now introduce the different S-gene signatures of the variants in the *Introduction* section, as well as a brief timeline of the different variants over time. The *Results* section repeats the periods studied and the S-gene results each variant corresponds to.

- If spatial variation is a focus then maps of the area/outcomes should be presented.

  Authors: The case distribution map figure (*"Omicron COVID-19 cases in Scotland (top) between..."*) has been promoted from the supplementary information to the main text. In the Supplementary Information we include a map of the population distribution of Scotland at DZ level, and a new figure showing spatial distribution of residuals.

- Both data zones and local authorities are discussed – why move between the two?

  Authors: We use datazones (6,976 total) as they are highest spatial resolution available to us. We also discuss at local authority level (32 total) as it is a more reasonable scale from which to calculate reproduction numbers and show regional variation. Further, in the context of the epidemic in Scotland, the local authority scale is important as this was the level at which NPIs were imposed and varied during the periods the study focuses on. We have stated this in the *Introduction* section and re-iterated it on discussion of R_t in the *Results* section.

- Figure 1 shows cases and hospitalisations – is the analysis repeated for both? I don't think this has been mentioned.

  Authors: The analysis is not repeated for both. Briefly, the much lower volume of hospitalisations makes a regression identical to the method used for cases less suitable due to a very large zero-inflation in hospitalisations at cohort level.

  Further, in acknowledgement of this point and a second reviewers' feedback, we have opted to remove the hospitalisations data from this figure and some brief discussion in the *Results* section, to simplify the presentation of case data and our model performance.

- Reference to calculating Moran's I which is not described in the methods.

  Authors: We have now defined the Moran's I formula explicitly in a new section in the *Data and methods* section: *"Moran's I autocorrelation statistic"*.

- The results section generally includes a lot of description of methods which should be in the methods section.

  Authors: We have re-organised the *Results* section and moved all description of methods to the *Data and methods* section. We do keep some contextual description of the ALE methodology as they may be a newer concept to the reader, but also refer them to *Data and methods*.

Discussion

- The authors reference on multiple occasions covid "circulating in high volume" – I'm not sure I know what you mean by this.

  Authors: We have replaced "circulate" with more precise terminology throughout the manuscript.

- There is a lot of literature on risk factors for infection/mortality throughout the pandemic which has not been referenced. I don't agree that there is "increasing uncertainty" in who is at highest risk – as information has accumulated over time our understanding has improved.

  Authors: We agree that the broad risk factors are clearly well understood, especially for severe outcomes. This point was more in the context of not understanding the precise patterns or being able to monitor the proliferation of novel variants – especially with changing levels of vaccination/infection-based immunity. We have rephrased the opening paragraph accordingly and made more references to the existing literature on risk factors.

- "Any differences as were observed are as likely to be explained by the differences in imposed interventions as they are due to differences in the virus strains." – this conclusion is not supposed by anything presented here.

  Authors: We have replaced this with the more precise statement:

  "We presented the accumulated local effects (Fig. 4), revealing broad indicators for higher or lower case rates, and how they changed between waves. It is difficult to fully disentangle whether a change was caused by a change in control measures, or a change in virus strain. Nonetheless, ...".

  This now sits in the subsection *Risk factors*, and we support the point with specific examples from the ALEs.

- Reference to "individual level" drivers but as far as I can tell the model was fit to aggregated observations ("cohorts")

  Authors: The reviewer is right to raise this ambiguity, and we have rewritten the paragraph.

  We fit to cohort-level and not individual. The individual causative drivers (who interacts with whom) are clearly beyond the reaches of the data/model. However our analysis helps to disentangle the contribution of factors that have high within-community variation (such as vaccination uptake, which can vary sharply from neighbourhood to neighbourhood), that may otherwise be obscured at a coarser resolution.

- "local outbreak duration parameter" – this hasn't been mentioned anywhere prior as far as I can tell

  Authors: This was defined in the supplementary material, but it should indeed be in the main text. It is now mentioned explicitly in the section *Explanatory variables.*

- Reference to "sampling" of cohorts but methods just says that cohorts were defined by grouping observations, not sampling from some wider pool.

Authors: This was meant in the context of the random forest fit – for each tree (on which the random forest fit is an average over many trees), cohorts (or more generally rows in the training set) are sampled with replacement. This negates overfitting. Then, by weighting that sampling in some way (as we did with population, so higher-population cohorts were selected more often), one can often achieve a better fit. We have removed one ambiguous mention of "sampling" in the *Discussion*, and clarified a second.

Reviewer 2

The manuscript "Assessing the importance of demographic risk factors across two waves of SARS-CoV-2 using fine-scale case data" by Wood et al. provides a comprehensive analysis of regional variations in SARS-CoV-2 spread using fine-scaled data on cases combined with various epidemiological and socio-economic factors. While none of the individual results presented in the paper are new by themselves, the study gives a quite comprehensive picture that allows one to better understand the importance of some of the factors in relation to other risk factors. The authors achieve a remarkable fit between their model and data, suggesting that the risk factors covered in these factors indeed capture most of the variation in regional spread. I just have a few minor comments aimed at improving the clarity and interpretability of the results.

- Overall, figures are sometimes hard to read due to the extremely small font size.

  Authors: We have revised many of the figures to have larger font sizes. Fig.1 (overall summary plots of case rates) was a particularly dense figure, and as discussed elsewhere in the feedback we have since removed the hospitalisations data, making the figure simpler.

- Regarding Fig. 2 and its caption, I'm not sure that the meaning of the results conveyed here are easily accessible to non-technical experts. If I understand it correctly, the message is that close-by regions (in two different senses) tend to show similar deviations with respect to the model, meaning that the unexplained variance is (at least in parts) due to regional influences? Maybe something along these lines can be stated more clearly. I was also wondering whether such an effect can also be seen by coloring the points in a scatter plot of data vs full model in a way that encodes administrative divisions/regions, or showing centroids of points belonging to the same region and some measure of their spread?

  Authors: We believe the reviewer is referring to Figure 3 here (in referring to proximity of regions / "two different senses" being spatial/network distance).

  Yes, the Moran's I plot illustrates how the residuals are correlated with separation. For example, for the nearest-neighbours plot, the Omicron Moran's I for 5 nearest-neighbours is about +0.1, thus the correlation between residuals when only looking at those within 5 nearest neighbours is +0.1.

  We have added a full description of the statistic in the *Materials and methods* section (subsection *Moran's I autocorrelation statistic*). For the Omicron model we have added map plots showing the residuals over space in the Supplementary Material, to illustrate the spatial autocorrelation.

- The differences in scales in Fig. 4 are a bit unfortunate but I understand their necessity. To make this clearer, one could maybe put population, age, sex, prior test on the same scale within one row and a bit detached form that the other variables, also all on the same scale. Furthermore, some of the factors with small ALEs that are also not really discussed at any point in the manuscript could be moved to the SI entirely.

  Authors: We have separated those four variables and placed them on a separate row, so the reader can more clearly see the difference. As we discuss further in the next comment, we keep all ALE plots in the main text as they form an important discussion point (and do indeed discuss them more now in a separate subsection of the *Discussion*).

- Speaking of which, what was the rationale behind the inclusion of some of the risk factors that show no ALEs and are not really discussed? What does this add to the study? Also negative results should be discussed.

  Authors: We see deprivation and sex as two interesting "negative" results (negative in the sense that their ALEs were weak). The reason for their inclusion was because they were highlighted as risk factors for severe outcomes elsewhere in the literature. The lack of variation in case rates seen in the data/model is then not obvious. It suggests a combination of 1) a heightened rate of severe outcomes *per case* for males/higher deprivation (which is of course well studied),

2) the case data not being wholly representative of the profile of all infections, due to variation in the rate of case ascertainment across different demographics.

We agree that this was not discussed enough in the initial manuscript to justify their inclusion. To address this we have done additional work (subsection *Testing frequency*), looking at rates of lateral flow testing (voluntary, self-reported) broken down by sex and deprivation. We discuss that lower testing rates were observed in males/more deprived DZs as well as higher positivity. Thus male/more deprived communities may have experienced more infections, but with fewer infections finally resulting in a positive PCR test. More generally we now assert in the *Discussion* section that analyses of reported cases need to be viewed with these strong skews in testing behaviour in mind.

- Some discussion on what are possible risk factors that account for the unexplained variance might be informative. Potential differences in non-pharmaceutical interventions, mobility and meteorological factors come to my mind based on previous literature: `https://doi.org/10.1016/S1473-3099(20)30553-3`, `https://doi.org/10.1371/journal.pcbi.1009973`, `https://doi.org/10.1073/pnas.2019284118`

  Authors: We agree that the three factors are likely causative risk factors for cases that have not been included in the model. We suspect a mobility is a key "missing" element – be that via transport links, school catchment areas etc. The model as set up is not informed by which datazones neighbour one another, whereas in reality case rates in one DZ are inevitably affected by those in neighbouring DZs. We have specifically discussed mobility and meteorological differences into the *Discussion* section, and referred to more related studies in the *Introduction*, including the three papers the reviwer notes here.

Reviewer 3

The Authors used a random forest model to assess the impact of various factors such as age, sex, prior infections and deprivation status on the risk of COVID-19 infection (variants Delta and Omicron). A definite strong point of the work is the unique dataset, comprising detailed, high-resolution spatial and demographic information about cases and hospitalizations in Scotland. The study is well-constructed, the hyperparameters are set to avoid overfitting, and the models are validated on an independent set. The results show that the risk factors were fairly consistent between the variants, which is an interesting observation.

Although the majority of features used in the model can be considered independent variables, my one concern is that the local outbreak duration was chosen as a proxy for spatial relationships between data zones. Particularly in densely populated areas, where data zones are likely more arbitrary and highly similar, the intermediate zone can be indicative of the duration in the considered zone itself. Since the outbreak duration depends on the number of cases, it is a derivative of the response variable, rather than a predictor. Indeed, both the ALE and model accuracy loss analyses showed high importance of this variable. Furthermore, since this information can only be obtained after the outbreak, inclusion of this variable limits the applicability of the proposed model for potential future COVID-19 waves.

Authors: We defined the local outbreak duration at intermediate zone level (containing ~ 6 DZs and forming more well defined communities relative to DZs) as once a case has been detected within the IZ, with the $\sim$ 5day incubation period and the considerable case under-ascertainment it is reasonable to assume that the variant is circulating amongst those well-connected DZs surrounding. In other words, a risk factor for COVID-19 in one area may be the presence of COVID-19 cases in communities surrounding it. For example, a high case rate in an otherwise "low-risk" area may simply be explained through early seeding and circulation in a neighbouring community.

We do agree with the reviewer that this will correlate to a degree with cases, and for future analyses of case distributions over shorter time periods, the staggered seeding of the variant across different DZs could be addressed more explicitly; for example, calculating cases/person/day, only counting days after first local detection of the variant. We included it here to account for connectivity between neighbouring DZs in the absence of mobility data – the rate of COVID-19 cases in a DZ are undoubtedly linked to the case rates in neighbouring DZs, thus the time the variant was detected in the broader vicinity (but not in the precise DZ) is important.

We suspect that this was less of a factor for Omicron (where the geographic spread was rapid and the variant was seeded in nearly all regions over $\sim$10 days).

We have added a new paragraph acknowledging these limitations in the *Discussion* section.

While I found the article a compelling read, I would also like to point out several technical issues:

- In line 69, please specify the year for both dates, since it is not the same.

  Authors: We have corrected this.

- In Fig. 1, please extend the figure description for a clearer perception - please specify what the numbers are relative to, perhaps include the N for the whole population. Moreover, the layout for the prior cases panel is different from the rest. Were no previously infected individuals hospitalized?

  Authors: We have revised the caption for this figure to make it more comprehensive, and included the overall population. For each wave the "prior cases" population is given as those that had tested positive within six months / over six months from the start of the wave. These will be different for each wave; for example more individuals had reported a case between 15th May and 15th Nov 2021 (taken as the "start" of the Omicron wave in the analysis) compared to between 1st Nov 2020 and 1st May 2021 (Delta).

  In light of feedback from other reviewers and on reflection we have decided to omit the hospitalisations data from this figure as well as a brief discussion later in the manuscript; on reflection we feel it distracts from the main narrative of the manuscript (to analyse variation in COVID-19 case rates between waves, and model performance), especially when we do not perform any further analyses on hospitalisations.

- In Fig. 2 and Supplementary Fig A.3 (B), the x axis tick labels overlap slightly.

  Authors: These have been corrected.

- In line 100, please replace " 'univariate' " with a more precise term - perhaps "reduced".

  Authors: We have replaced "univariate" with "reduced" and described these models more clearly in the *Random forest model* section.

- Particularly in the Results section, the descriptions tend to be slightly chaotic - for example, lines 169-174 would be more fitting for the "Detailed case distribution analysis" subsection.

  Authors: We have made a significant revision to the *Results* section so as to move a lot of text better suited for the *Data and methods* and *Discussion* sections. We hope this makes the *Results* section much clearer.

- From the description, it seems that the model hyperparameters were set arbitrarily. Please avoid using the word "optimised", "maximises" etc. unless an optimisation procedure was performed. If different hyperparameter values were indeed tested, the results would be a valuable addition.

  Authors: We did test a variety of hyperparameters to specifically minimise the performance difference between the training and test data. This was a manual procedure, which we now state in the main text.

- For non-standard abbreviations (eDRIS, DZ) please include the explanation not only in the Materials and Methods section, but also at the first use - it would improve readability.

  Authors: We now define DZ and eDRIS on first use in the *Introduction* section. This arose on moving the *Data and Methods* section to the end of the main text.

- Please ensure that all of the references adhere to the required format and are consistent - for example, in reference 28, the Office for National Statistics is mentioned after the title, and in reference 31 before, as "for National Statistics, O". For online resources, please include the access date, or, in case of R packages, the version number.

  Authors: We have corrected the erroneous formatting. R and associated package versions are now stated, and we have checked the links of all online resources and stated a date of last access.

Reviewer 4

The authors of the article, among others, built a model using random forests to predict the distribution of cases in Scotland. They applied modeling to two successive waves (Delta, Omicron) of the Covid-19 pandemic. They concluded that the distribution of cases could, to some extent, be explained by independent variables such as age, deprivation and population density. In addition, they indicated that the detected relationships remain largely unchanged regardless of which wave of the Covid-19 pandemic was analyzed.

The presented results look very interesting. Unfortunately, the authors did not take care to explain in detail how the predictive model was built and what data it was supplied with.

Major comments.

- The authors write that they consider the so-called geographical data zones (DZs) covering about 1000 inhabitants each. As a reviewer, I understand this to mean that from a machine learning (or statistical) point of view, one data zone constitutes one pattern (observation). This is suggested by Figure 2, in the description of which it is indicated that one point corresponds to one DZ. The problem is that the authors have not clearly described what the explained and explanatory variables are. I do not understand what is on the axes of the graphs shown in Figure 1. The axes are marked with the word "Cases" - what does it mean? Number of Covid-19 cases in one data zone? The range on these axes is 1-100. Is it a percentage number of cases in one DZ?

  Authors: An "observation" in this context is the number of cases identified from a cohort (refering to the main text, an example cohort could be males aged between 50-54 living in datazone "X" that had never tested positive for COVID-19 before, with a population of 38 and amongst whom 9 cases were identified). We have added to the *Data and methods* section to clarify our definition of a "cohort" with an illustrative example, and how we aggregate these cohorts in different ways to produce our fit value for coarser measures e.g. Local Authorities, or individuals living in Remote Rural communities.

  The axes in Figure 1 are the total number of cases in each DZ in that period (and our fit value). We have updated the axis labels.

- A more serious problem is the explanatory variables. While deprivation or population density, for example, are obvious, it is not entirely clear what the "Age" variable means for a given data zone. If there are about 1000 inhabitants in one DZ, does "Age" mean average age or median age? If so, it seems that the variable "Age" defined in this way should be more or less constant for different areas of Scotland. Given this, it is strange that a relatively good match was achieved for "Age", comparable to "Deprivation" or "Density". It is possible that this has something to do with the "Accumulated local effects" described in section 2.2.1. If so, it should be clearly stated.

  Authors: We clarify this in the (new) section *"Preparation of case data"*. Here we describe that while the spatial resolution of the data is at DZ level, as we know the explicit age and sex of each individual in the test data, we can divide the data further by sex/age range (then one further division, based on whether individuals have tested positive in the past or not). Thus each of the 6,976 DZs has 2 (sex) $\times$ 16 (age range) $\times$ 3 (tested positive in last 6 months, tested positive over 6 months prior, never tested positive before) cohorts. Each of cohort at this level has an age, which is fed into the model.

  As we know the exact age and sex, then, we should expect to achieve a good fit to these values. Whereas deprivation and population density are coarser (i.e. we do not know the deprivation status of every individual, just of their residing community, thus it may be a weaker explanatory variable), it is expected that the fit would not be as precise, but the model does capture the overall trend well. In the *Discussion* section we now discuss the model performance more and what we learn from the ALEs, particularly with respect to deprivation.

Minor comments.

- Does Figure 2 show the results for the training set or the test set?

  Authors: This is for both sets combined, which we now state explicitly. We quote the variance explained for both sets in the main text, however unfortunately as our data access agreement has since expired we can not re-run the model to produce set-separated plots.

- The abbreviation "DZ" was not explained anywhere and as a reviewer I had to guess it.

  Authors: We now define DZ (datazone) on first use in the section *Introduction*. This arose on moving the *Data and Methods* section to the end.

- The authors wrote in the abstract that One DZ covered about 1000 inhabitants. And elsewhere, in the caption of Figure A4, it says 500-1000. What is the truth?

  Authors: This is indeed a discrepancy on our behalf; the range 500-1,000 is more accurate and we have corrected the abstract. (The mean DZ population is 786, with 82% having a population in the range 500–1,000, per the cited report `https://www.nrscotland.gov.uk/files//statistics/population-estimates/sape-2021/sape-21-report.pdf`).

- It would be interesting to build a model for the first Delta wave of the Covid-19 pandemic and use it to predict the second wave of Omicron.

  Authors: We agree – looking into the future the main risk factor that is likely to change most substantially is the level of immunity from infection/vaccination. A model built in a way similar to ours could be used to generate counterfactual scenarios more broadly.
